# Dynamic changes of postprandial plasma metabolites after intake of corn-soybean meal or casein-starch diets in growing pigs

**DOI:** 10.1186/s40104-019-0351-8

**Published:** 2019-05-28

**Authors:** Tiantian Li, Shimeng Huang, Juntao Li, Hu Liu, Wei Wang, Na Li, Meng Shi, Shiyu Tao, Shuai Zhang, Zhen Li, Junjun Wang

**Affiliations:** 1 State Key Laboratory of Animal Nutrition, College of Animal Science and Technology, China Agricultural University, Beijing, 100193 China; 2 State Key Laboratory of Plant Physiology and Biochemistry, College of Biological Sciences, China Agricultural University, Beijing, 100193 China

**Keywords:** Diet, Metabolomics, Plasma metabolites, Pig, Postprandial

## Abstract

**Background:**

Postprandial nutrients utilization and metabolism of a certain diet is a complicated process. The metabolic feature of pigs after intake of corn-soybean meal or casein-starch diets are largely unknown. Therefore, this study was conducted to investigate the dynamic postprandial changes of plasma metabolic profile using growing pigs using metabolomics.

**Methods:**

Twenty-four growing pigs with average initial body weight (BW) about 30 kg were placed in metabolic cages and then fitted with precaval vein catheters. Pigs were fed daily 4% of initial body weight. Two experimental diets were included: (i) a starch-casein based purified diet (PD) and (ii) a common corn-soybean meal diet (CD). Plasma was collected before feeding and 0.5 h, 1 h, 2 h, 4 h, 8 h after feeding.

**Results:**

In both diets, compared to prior to feeding, the concentrations of glucose, most amino acids, metabolites such as 5-aminopentanoic acid, pipecolic acid, ornithine and 5-hydroxy-*L*-tryptophan were significantly increased in plasma during the first hour, whereas the concentrations of plasma triglycerides, glutamate, glycine, palmitelaidic acid, 13-HODE and oleic acid were decreased in the first hour. Compared with PD group, concentration of plasma leucine and isoleucine declined at 30 min in CD group. Plasma linoleic acid, sphingosine and many dipeptides were significantly higher in pigs fed CD.

**Conclusion:**

Most significant metabolic changes occurred during the first hour after feeding and then became relatively stable after 2 h in both diets. These results show a broad scope picture of postprandial changes in plasma metabolites after intake of PD and CD and could be a reference for further nutrition intervention as well as the design of nutritional studies.

**Electronic supplementary material:**

The online version of this article (10.1186/s40104-019-0351-8) contains supplementary material, which is available to authorized users.

## Background

Nutrition is the provision of nutrients that are necessary to support and maintain life. Currently, nutritional science mainly investigates the physiological and metabolic response to different nutrients and focuses on improvements through nutritional (diet) intervention [1]. Meanwhile, the physiological and biochemical response to the intake of a certain diet are an inherently complicated process [2, 3]. Following the meal ingestion, dietary glucose and amino acids (AA) are transported and absorbed from the intestine into the blood, while the majority of lipids are packaged into chylomicrons and distributed to the tissues using the lymphatic system and have diverse physiological influence on the body [4]. Although complex to measure, the metabolic response to a nutritional challenge has been proven more useful in evidencing differences between healthy and pathological phenotypes than in the fasting state [5]. Postprandial metabolic profiles are also closely linked with physiological responses to a dietary intervention and some of the metabolites facilitated to understand the interaction and regulatory roles of nutrition [6]. Considering that glucose can be a metabolite used to design personalized diets [7], we suppose that other metabolites in blood could also be exploited as nutrition-related biomarkers.

Corn and soybean are the major crops globally. Corn is one of the most important ingredients in food and feed acting as the main energy source. Soybean meal, the byproduct of soybean oil, are widely used in feedstuff as protein source and contains specific nutrients, including isoflavone, saponin, and phytosterol [8]. At the same time, casein and starch are also used as protein and energy source for various purposes [9]. Dietary amino acid and energy supplied from casein and corn starch-based diets were conventionally used as purified diet to check the responses to nutrients intake [10].

The metabolome is defined as the collection and global analysis of all small molecule metabolites generated in a cell, organ, or organism [11]. Metabolomics can help tracking the interaction between nutrients and metabolism [12], and give us a better understanding of nutrients digestive and absorptive kinetics postprandially. The metabolomics study in mini-pig showed that many metabolites displayed opposite trends postprandially which could be a compensatory mechanism in response to the nutrient influx [5].

Therefore, the main objective of current study was to obtain a broad scope picture of postprandial metabolites changes after intake of PD and CD in growing pigs, which will increase our understanding of the digestive and absorptive kinetics for these two classic diets and provide basis for selection of time spots for blood sampling used for various purposes.

## Methods

### Diet, animals and experimental design

The animal care and experimental procedures were approved by the China Agricultural University Animal Care and Use Committee (CAU20161014–1). Detail of the two diets, (i) a starch-casein based purified diet (PD) and (ii) a common corn-soybean diet (CD), are shown in Table 1. Twenty-four growing pigs (Landrace × Yorkshire), with an average initial BW of 30.05 ± 0.87 kg, were selected from the Ministry of Agriculture Feed Industry Center Animal Testing Base (Hebei, China) and were individually placed into stainless-steel metabolism cages in the Laboratory of Animal Metabolism in Fengning Swine Research Unit of China Agricultural University (Hebei, China). Pigs were adapted to the cages and the catheters for 7 d prior to the start of the experiment, and then they were fed either of the experimental diets (12 for PD, 12 for CD) for 5 d before blood sample collection. Pigs were weighed at the beginning of the experiment and fed twice daily according to their body weight (total 4% of initial body weight daily) at 08:00 and 17:00 h. Pre-experiments were done to ensure the amount of feed for morning feeding so that pigs can finish eating within 40 min. The same amount of feed was given in the afternoon. Feed refusals and spillage were recorded daily. Water was freely available from low-pressure drinking nipples.

**Table 1:** Composition of the experimental diets (%, as-fed basis)

Items	Corn-soybean meal based diet (CD)	Starch-casein based purified diet (PD)
Corn starch	0	72.85
Corn	70.85	0
Casein	0	19
Soybean meal	25	0
Dicalcium phosphate	0.6	0.6
Limestone	0.7	0.7
Salt	0.35	0.35
Acetyl cellulose	0	4
Soybean oil	2	2
Vitamin and mineral premix^a^	0.5	0.5
Total	100	100

^a^Premix provided the following per kilogram of complete diet for growing pigs: vitamin A, 5512 IU; vitamin D_3_, 2200 IU; vitamin E, 30 IU; vitamin K_3_, 2.2 mg; vitamin B_12_, 27.6 μg; riboflavin, 4 mg; pantothenic acid, 14 mg; niacin, 30 mg; choline chloride, 400 mg; folacin, 0.7 mg; thiamin 1.5 mg; pyridoxine 3 mg; biotin, 44 μg; Mn, 40 mg (MnO); Fe, 75 mg (FeSO_4_·H2O); Zn, 75 mg (ZnO); Cu, 100 mg (CuSO_4_·5H_2_O); I, 0.3 mg (KI); Se, 0.3 mg (Na_2_SeO_3_)

### Surgery

Each pig was fitted with a catheter in the anterior vena cava via the cephalic vein and the main surgery procedure were as described in detail by Takken et al. [13]. Simply, anesthesia was induced by intramuscular injection of 5 mg/kg Zoletil 50, then pierce the skin of surgical site (crossline of point of sternum, jugular furrow and cephalic veins) using a hollow needle with guide wire, then pull out the needle and insert the catheter along the guide wire. Pull out the guide wire and fix the catheter with butterfly clip. The catheter was blocked with a heparin solution and the pig was given intramuscular injection of antibiotic (Cefazolin) at 50 mg/kg.

### Blood sampling

Blood samples of each pig were collected at d 5 after start of the experimental period. Baseline sample were collected 10 min before morning feeding and then blood samples were collected 30, 60, 120, 180, 240 and 480 min after feeding. Samples were centrifuged (Biofuge22R; Heraeus, Hanau, Germany) at 3,000×*g* for 10 min, then the supernatant was transferred into other tubes and frozen in liquid nitrogen, then stored at − 80 °C for metabolomics analysis.

### Chemical analysis

The feed samples were ground to pass through a 1-mm screen and mixed thoroughly for chemical analysis (Table 2). All chemical analyses were conducted in duplicate. Samples of ingredients and diet were analyzed for dry matter (DM) and crude protein (CP) [14]. Amino acid composition of diets was determined according to the procedures of Association of Official Analytical Chemists (AOAC) (2000). Samples were hydrolyzed before analysis with 6 mol/L HCl for 24 h at 110 °C and analyzed for 15 AA with an Automatic Amino Acid Analyzer (L-8900 Hitachi Automatic Amino Acid Analyzer, Tokyo, Japan). After cold performic acid oxidation overnight and hydrolysis with 7.5 mol/L HCl for 24 h at 110 °C, methionine and cystine were analyzed as methionine sulfone and cysteic acid. Tryptophan was determined after LiOH hydrolysis for 22 h at 110 °C by High Performance Liquid Chromatography (Agilent 1200 Series, Santa Clara, CA, USA) [15].

**Table 2:** Amino acid profile of the experimental diets (%, as-fed basis)

Items	Corn-soybean meal based diet (CD)	Starch-casein based purified diet (PD)
Crude protein	17.22	17.24
Lysine	0.90	1.36
Methionine + Cystine	0.63	0.60
Threonine	0.68	0.77
Tryptophan	0.24	0.26
Isoleucine	0.72	0.89
Leucine	1.65	1.64
Valine	0.85	1.16
Arginine	1.17	0.63
Histidine	0.49	0.50
Phenylalanine	0.90	0.90

### Biochemical analyses

The plasma glucose concentration was determined by ion-exchange chromatography (Agilent 1200). Briefly, samples were clarified by centrifugation at 12,500 × *g* for 10 min at 4 °C, then supernatants (0.25 mL) were diluted to 5 mL with water and passed through a hydrophobic filter cartridge before analysis. Plasma insulin concentration was examined using porcine insulin INS ELISA Kit (MSK). Triglyceride, plasma calcium, ALB, HDL, LDL and plasma urea nitrogen were analyzed using Hitachi 7020 Chemistry Analyzer according to Wang et al. [16].

### Amino acids profiling based on LC-MS/MS

Three hundred μL plasma was pipetted into a 2-mL centrifuge tube, 12 μL of internal standard (2.5 mmol/L norleucine) was added, and 1.2 mL ice-cold methanol was added. After vortex, the samples were centrifuged at 14, 000 × *g* for 10 min at 4 °C (Eppendorf, Hamburg, German). A 500-μL supernatant were evaporated to dryness in a vacuum concentrator (Eppendorf, Hamburg, German). Residues were resuspended in 100 μL of borate buffer (AccQ-Tag Ultra Derivatization Kit, Waters, USA), vortex-mixed and centrifuged again at 14,000 × *g* for 10 min at 4 °C. A 10-μL aliquot of the supernatant was then mixed with 50 μL borate buffer in a vial. Then, 20 μL derivatization reagent (AccQ-Tag Ultra Derivatization Kit, Waters, USA) dissolved in acetonitrile was added. Vials were capped, vortex-mixed, and heated to 55 °C for 10 min. After cooling to room temperature, the solutions were transferred to sampler vials for LC-MS/MS analysis. Standard solutions were derived in the same manner. The LC-MS/MS was performed according to previous study [17, 18].

### Metabolomics measurement based on UPLC-MS

Plasma samples from 12 pigs of each treatment were extracted using 800 μL of ice-cold extraction mix (acetonitrile: methanol, 1:1, *vol*: *vol*) at 1:4 sample: extraction mix ratio. After 5-min vortex, the samples were centrifuged at 18,000 × *g* for 10 min at 4 °C for deproteinization. Then, the supernatant fractions were collected and evaporated to dryness using a vacuum concentrator (Concentrator plus, Eppendorf). The resulted dry residues were re-suspended in 200 μL of 50% methanol, vortex-mixed and centrifuged again at 18,000 × *g* for 10 min at 4 °C. At last the supernatant fractions were filtered through a 0.1-μm membrane (Millipore, USA) and transferred to sampler vials to be analyzed on the LC-MS system.

Plasma samples were analyzed with an UPLC-MS system (UPLC, Ultra Performance Liquid Chromatography, ACQUITY UPLC H-Class Bio, Waters; MS, Mass Spectrometry, Q-Exactive, Thermo Scientific) equipped with a heated electrospray ionization (HESI)source. UPLC separation was operated on a BEH C18 column (2.1 mm× 100 mm, 1.7 μm, Waters). Mobile phase A: 0.1% formic acid water solution; B: 0.1% formic acid acetonitrile solution (all HPLC grade, Thermo Fisher Scientific, NJ, USA). The gradient program as follows: 95% A at 0 min to 70% A at 5 min, 5% A at 10 min and held for 3 min, then returned to initial condition. The flow rate was set at 0.3 mL/min. A sample of pooled plasma and blanks was re-injected after every six samples for quality control. The column temperature was set at 35 °C and the injection volume was 5 μL.

MS analysis was performed in an electrospray ionization positive mode. Full scan data was acquired with a resolution of 70,000 in the mass range of *m/z* 67.7–1,000. In addition to full-scan acquisition, tandem mass spectrometry (MS/MS) analysis was carried out using a mass inclusion list which included *m/z* and retention times of targeted differential metabolites, an isolation window of 0.8 *m/z* and a mass resolution of 35,000 were selected. For MS/MS analysis, collisions were performed at the energies of 35 V.

### Analysis of metabolomics data

SIEVE 2.1 software (Thermo Scientific) was used for metabolomics data processing. The software picks out peaks beyond a preset threshold, subtracts the background and extracts component from the raw data. Filtering of the compounds with CV < 30%, fold change > 1.5 and *P* < 0.05 was performed using Excel for further identification. Identification of differential compounds was performed in compound database of METLIN (https://metlin.scripps.edu/) and the Human Metabolome Database (http://www.hmdb.ca) using accurate mass of molecular ions. MS/MS spectra database was used to match fragment ion spectra of the candidate compounds. MS/MS spectra were also compared with theoretical fragmentation pattern with mass accuracy at 5 ppm using Xcalibur™ (Thermo Scientific). Metabolite identifications can be classified as level II based upon their spectral similarities with public/commercial spectral libraries in accordance with the MSI guidelines [19]. The differences among means were determined by Tukey’s multiple range test. Results were considered significant at *P* < 0.05 and as trend at 0.05 < *P* < 0.1. Fold change was calculated by dividing the mean of normalized intensity of each plasma metabolite in the former by the mean of normalized intensity of each plasma in the latter. Fold change > 1 indicates that the metabolite was up-regulated, whereas fold change < 1 indicates the metabolite was down-regulated.

### Statistical analyses

The differential metabolites of PD and CD within different time spots were analyzed by SAS (version 9.2) using repeated measures analysis of variance. Boxplot analysis of identified differential compounds were achieved using the R software package (R Development Core Team (2017), version 3.4.1). Pathway analysis of metabolite profiles was carried out using MetaboAnalyst 4.0 (http://www.metaboanalyst.ca).

## Results

### Postprandial energy metabolism

**Fig. 1:**
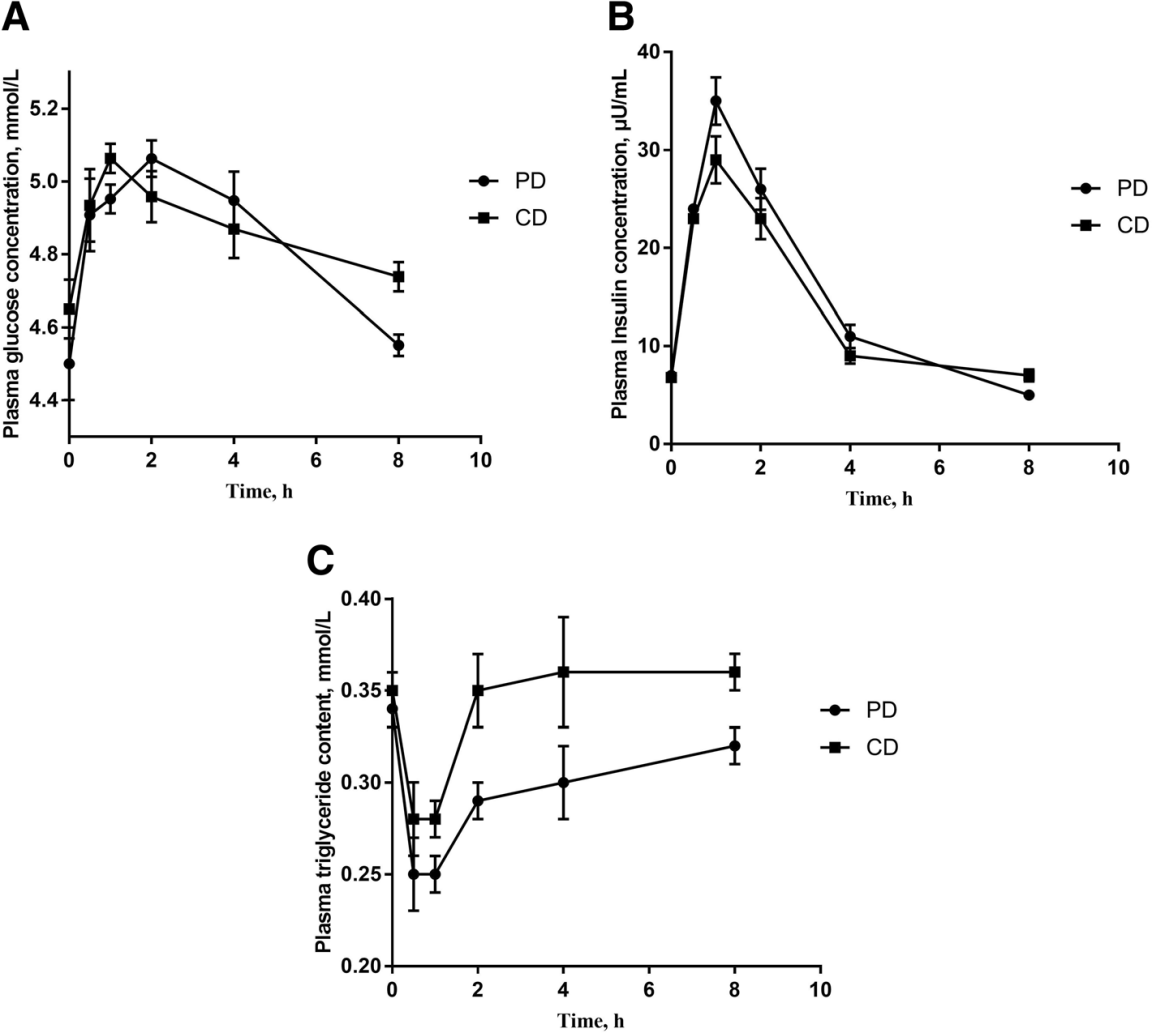
Dynamic changes of plasma concentration of glucose (**a**), insulin (**b**), and triglyceride (**c**) after intake of PD and CD. PD: starch-casein based purified diet; CD: common corn-soybean meal based diet. The plasma glucose concentration of PD was higher than of CD at 2 h (*P* < 0.05). Plasma insulin concentration of PD was higher than of CD at 1 h (*P* < 0.05). Plasma triglyceride content was lower in 30 min and 1 h than 2 h of both diets (*P* < 0.05). PD: a starch-casein based purified diet and CD: a common corn-soybean meal diet

Plasma glucose concentrations of PD and CD (before meal and 0–8 h after meal) are presented in Fig. 1a. The plasma glucose concentration increased significantly at 30 min in both diets (*P* < 0.05), peaked at 1 h in CD group then decreased significantly at 2 h (*P* < 0.05). In the PD group, the plasma glucose concentration gradually increased and peaked at 2 h (*P* < 0.05). The insulin response showed a similar trend in both groups (Fig. 1b). Plasma triglyceride content of two group were similar, which were lower in 30 min and 1 h than before meal (Fig. 1c).

### Postprandial amino acid changes

**Fig. 2:**
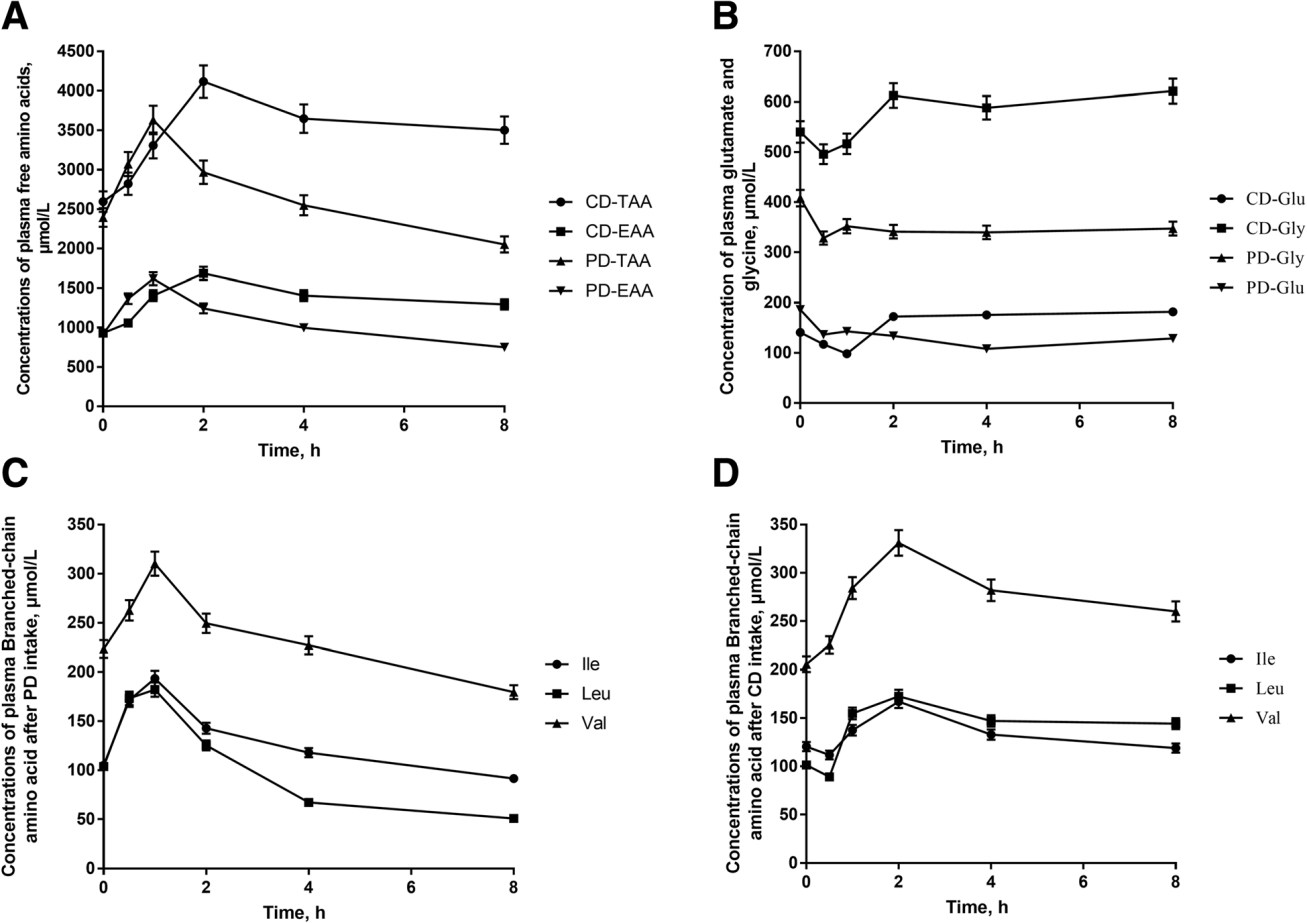
Concentrations of plasma free amino acids (**a**), glutamate and glycine (**b**) after intake of PD and CD, concentrations of plasma branched-chain amino acids after PD intake (c) and branched-chain amino acids after CD intake (**d**). PD: starch-casein based purified diet; CD: common corn-soybean meal based diet. TAA: total amino acid; EAA: essential amino acid. TAA was higher in CD than PD at 2 h (*P* < 0.05). Compare to baseline (10 min before morning feeding) glutamate and glycine significantly decreased at 30 min after intake of both diets (*P* < 0.05). Plasma branched-chain amino acid after PD intake showed a similar trend with TAA and EAA of PD. Compare to baseline, leucine and isoleucine decreased at 30 min (*P* < 0.05), increased and peaked at 2 h after CD intake, but valine showed a similar trend of TAA and EAA of CD. PD: a starch-casein based purified diet and CD: a common corn-soybean meal diet

Plasma free AA of PD and CD were examined. The concentration of total amino acid (TAA) and essential amino acid (EAA) after PD intake significantly increased and peaked at 1 h (*P* < 0.05). However, the concentration of TAA and EAA after CD intake showed a constant increase and peaked at 2 h and was much higher than that after PD intake (*P* < 0.01). Also, the concentrations of TAA and EAA after CD intake was higher at 8 h than before feeding (*P* < 0.05) while the concentrations of TAA and EAA after PD intake was lower at 8 h than before feeding (*P* < 0.05) (Fig. 2a). Most of the amino acids showed a similar trend with TAA and EAA except glycine and glutamate (Fig. 2b). Compared with that at 0 h (before feeding), both glycine and glutamate concentration in plasma significantly decreased at 30 min after PD intake (*P* < 0.05) and at 30 min and 1 h after CD intake (*P* < 0.05). Afterwards, the plasma glycine and glutamate concentration were higher at 1 h after PD intake and peaked at 2 h after CD intake, which were the same time points as TAA and EAA’s. The branched-chain amino acid showed a similar trend with TAA and EAA after PD intake (Fig. 2c), but after CD intake, the leucine and isoleucine decreased at 30 min (*P* < 0.05), then increased and peaked at 2 h (Fig. 2d).

### Other plasma metabolites changes

**Fig. 3:**
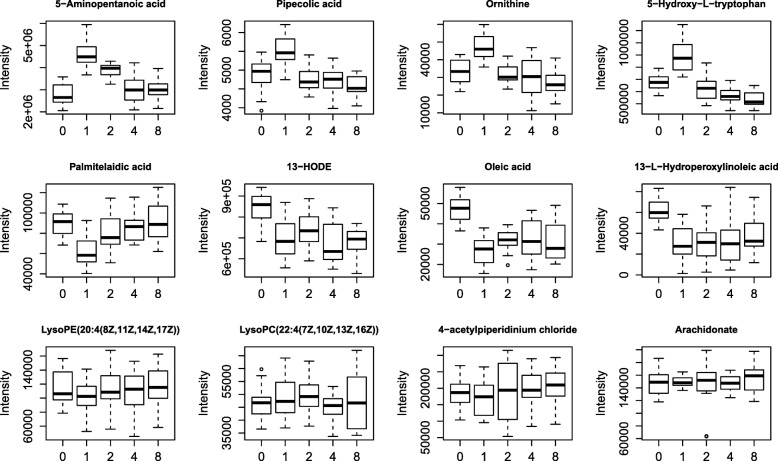
The differential metabolites whose relative abundance above 1 × 10^5^ at different time points after intake of both diets. 5-aminopentanoic acid, pipecolic acid, ornithine and 5-hydroxy-L-tryptophan significantly increased in first 1 h (*P* < 0.05), while palmitelaidic acid, 13-HODE,13-L-hydroperoxylinoleic acid and oleic acid, were lower in first 1 h than before feeding (*P* < 0.05). To note, 4-acetylpiperidinium chloride, lysophosphatidylethanolamin (lysoPE, 20:4(8Z,11Z,14Z,17Z), lysophosphatidylcholine (lysoPC, 22:4(7Z,10Z,13Z,16Z)) and arachidonate remained relatively stable at all the timepoints. PD: a starch-casein based purified diet and CD: a common corn-soybean meal diet

**Fig. 4:**
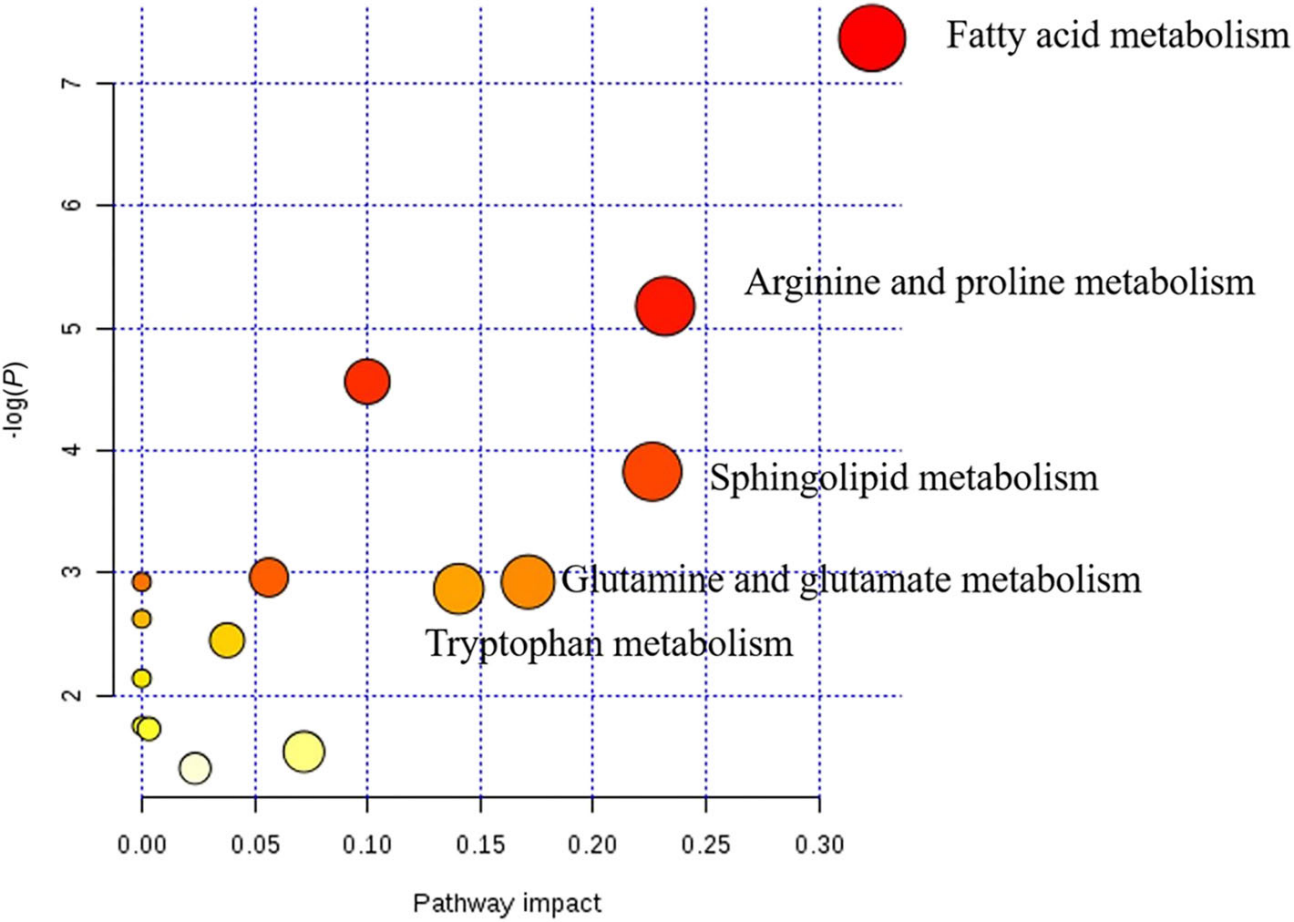
Pathway analysis generated with MetaboAnalyst 4.0 based on differential metabolites from CD vs PD. Node radius size and importance (*X-* axis) reflects the pathway impact values calculated using betweenness centrality, which takes into consideration the global network structure and measures the number of shortest paths going through metabolites within the node. Node color and direction (*Y*-axis), however, is based on the calculated *P* value of the enrichment analysis. PD: a starch-casein based purified diet and CD: a common corn-soybean meal diet

There were no significant differences of plasma phosphorus, ALB, HDL, LDL and CREA between PD and CD (Additional file 1: Table S1). As for the metabolomics results, 80 differential metabolites were identified according to secondary mass fragments and database (Additional file 2: Table S2). Among them, 12 metabolites whose relative abundance were above 1 × 10^5^ were visualized in the form of box-plot to show the direction of changes at different time points (Fig. 3). Most of these metabolites significantly increased postprandially, in consistent with the trend of TAA and glucose. 5-aminopentanoic acid, pipecolic acid, ornithine, 5-hydroxy-*L*-tryptophan, 2-ethylacrylic acid and 4-amino-2-methylenebutanoic acid significantly increased in 1 h (Table 3), while palmitelaidic acid, 13-HODE,13-L-hydroperoxylinoleic acid and oleic acid, were lower in 1 h than before feeding. To note, 4-acetylpiperidinium chloride, lysophosphatidylethanolamin (lysoPE, 20:4(8Z,11Z,14Z,17Z), lysophosphatidylcholine (lysoPC, 22:4(7Z,10Z,13Z,16Z)) and arachidonate remained relatively stable at all the timepoints. The identified differential compounds were related to different nutrient metabolisms: fatty acid, amino acids and their derivatives, peptides and other metabolism. Pathway analysis are shown in Fig. 4. As for relative abundance, most amino acids and their derivatives showed a similar trend of TAA in either treatment, especially the metabolites related to valine, serine and threonine metabolism. All the identified dipeptides such as *L*-leucyl-*L*-proline, *L*-gamma-glutamyl-*L*-leucine and serinyl-gamma-glutamate were found significantly up-regulated in CD group compared to PD group. The abundance of metabolites related to linoleic acid metabolism and sphingolipid metabolism were also significantly up-regulated after CD intake than PD intake.

**Table 3:** Identified metabolites with significant differences after intake of two diets in first 1 h

Description	MS^a^	RT^b^, min	0.5 h vs before meal		1 h vs before meal	
			Fold change	*P*-value	Fold change	*P*-value
*D*-Proline	115.1305	0.92	3.61	< 0.01	2.81	< 0.01
5-Aminopentanoic acid	117.1463	1.05	3.26	< 0.01	1.63	< 0.01
Pipecolic acid	129.1570	1.11	1.22	0.02	1.14	0.04
Ornithine	132.1610	0.80	1.71	< 0.01	1.41	< 0.01
5-Hydroxy-L-tryptophan	220.2246	2.66	1.94	< 0.01	1.32	< 0.01
Octadecanedioic acid	314.4602	10.25	1.88	< 0.01	2.23	< 0.01
Palmitelaidic acid	254.4082	8.65	0.62	0.03	0.70	0.04
13-HODE	296.4449	10.92	0.58	0.02	0.42	< 0.01
Oleic acid	282.4614	11.04	0.48	< 0.01	0.56	< 0.01
13-*L*-Hydroperoxylinoleic acid	312.4443	9.79	0.27	< 0.01	0.47	< 0.01

^a^M-to-Z ratio. ^b^rentention time

## Discussion

The physiological and biochemical response to the intake of a certain diet are a complicated process. The results of postprandial plasma metabolomes are important for revealing the digestive and absorptive dynamics of nutrients. The main aim of the present work was to investigate the postprandial changes of plasma metabolites using metabolomics combined with targeted analytical approaches such as classical biochemistry and quantitative free amino acid profiling. PD and CD were used in the present study to investigate the dynamic changes after the intake of these two diets.

Plasma glucose concentration can be affected by the time of feeding, digestion rate and insulin response. In the current study, pre-experiments were done to ensure that pigs can finish eating within 40 min. The corn starch used in PD and corn used in CD are both rapidly digestible starch. The results showed that the plasma glucose concentration increased significantly from 30 min after feeding till 1 h. Unlike plasma glucose, triglycerides concentration had a decline at 30 min and 1 h after intake of both diets, which may correlate with the higher level of insulin at 30 min and 1 h. Another reason for the decline could be that, compared with starch, lipid is slowly digested by animals and unsaturated fatty acids, saturated fatty acids and glycerol are the main substrates used for biosynthesis of triglycerides [20]. So, the triglycerides concentration increased at 2 h and remained at later time point. Although the changes of postprandial metabolites shared similarities between the two diets, there were still some differences between the PD and CD intake. Plasma glucose concentration of PD peaked at 2 h, which was unexpected and inconsistent with previous studies concluding that the highest plasma glucose concentration was 30–60 min after feeding [21, 22]. The similar trend of plasma insulin concentration of two diets suggests that the insulin is not the main cause of delayed peak of plasma glucose after PD intake. One possible explanation is that the sticky starch-casein diet, especially casein, as a coagulating protein, could slow the ingestion and the rate of gastric emptying [23] and cellulose acetate lowered the passing rate of digesta in the small intestine [24]. Another explanation is that in some starch-rich experiments, both in pigs and human, the profile of glucose concentration also peaked at 2 h; in those experiments, glucose concentration increased at 1 h, then decline significantly at 1.5 h and finally peaked at 2 h [25]. The current study did not collect the sample at 1.5 h, that made the profile seemed a continuously increase after feed intake. Therefore, it appears that the dietary starch source can play key role in affecting the plasma glucose concentration.

AA composition of the protein sources in the diets, postprandial peripheral AA profiles are likely to reflect rates of digestion, absorption and first-pass metabolism in the enterocyte and liver. The concentration of TAA and EAA both increase significantly 30 min and 1 h in both diets. Interestingly, concentrations of plasma glutamate and glycine decreased significantly after feeding in both groups. A previous study showed that 95% enteral glutamate was metabolized in first-pass by the gastrointestinal tract of pigs while only 28% phenylalanine was metabolized before entrancing the portal vein [26], resulting in a peripheral lack when other amino acid catabolism boost. As for glycine, previous studies showed that when fed a corn-soybean meal diet, the metabolic rate of glycine and alanine in growing pigs in the liver were 30–40 mg/min, accounting for about 60% of amino acids metabolized in the porcine liver [26]. These partially explained why the glycine content declined at 30 min after feeding. The concentrations of TAA and EAA after CD intake was higher at 8 h than before feeding and 8 h of PD intake, implying a possibility for relatively shorter feed interval while feeding easily digestible diets like PD. The concentration of TAA and EAA after PD intake significantly increased and peaked at 1 h, which is in accordance with previous study [27]. However, concentrations of the TAA and EAA after CD intake peaked at 2 h, that may relate to the glucose changes. Studies indicate that glucose is an important signal molecule in regulating the AA transporters by mTOR and thus high level of glucose could increase the amount of AA absorbed into the systemic circulation [9]. Therefore, the concentrations of the TAA and EAA peaked after the glucose reached the highest level. In addition, we found that the concentration of leucine and isoleucine decreased at 30 min only after CD intake. This is not accord with previous study which showed that leucine from dietary protein can bypass first metabolism in the liver, the expected sharp rise of plasma leucine levels were showed after PD intake [28, 29]. That may relate to the higher leucine to lysine ratio of CD, study showed that excess dietary leucine increased the catabolism of BCAA [30]. Another possibility is that the valine to lysine ratio was higher in CD (0.94) than PD (0.85). Study showed that when the valine to lysine ratio was higher, the concentration of leucine and isoleucine was lower at 40 min after feed intake [31]. Because BCAAs share the same enzymes to compete for transamination and oxidative decarboxylation catalysis [30].

Combined with classical metabolic approaches, metabolomics provides full-scope and more in-depth information of the global metabolite profile of plasma [32, 33]. Lysine, valine, proline, tryptophan and their immediate precursor or derivatives such as 5-aminopentanoic acid, 4-amino-2-methylenebutanoic acid and 5-hydroxy-*L*-tryptophan were up-regulated and all these metabolites were related to pathways including *D*-arginine and *D*-ornithine metabolism, arginine and proline metabolism, lysine degradation, glycine, serine and threonine metabolism. These results are consistent with the amino acid profiling. Interestingly, palmitelaidic acid, 13-HODE, 13-*L*-hydroperoxylinoleic acid and oleic acid were much lower after feeding. 13-HODE are oxygenated products of linoleic acid which were found as one of the strongest oxylipin-marker [33]. These metabolites were all related to linoleic acid metabolism and may down-regulated due to slow absorption and strong energy metabolism. Among all identified metabolites, the concentration of 4-acetylpiperidinium chloride, lysoPE, lysoPC and arachidonate remained at a relatively stable level at all time spots, showing that they can minimize the timeline effects be used as potential internal references.

Metabolomics results also showed that several dipeptides such as alpha-dimorphecolic acid, aspartyl-isoleucine, aspartyl-leucine, *L*-beta-aspartyl-*L*-leucine, *L*-gamma-glutamyl-*L*-isoleucine, *L*-gamma-glutamyl-*L*-leucine and *L*-gamma-glutamyl-*L*-valine were found up-regulated after CD intake, especially in 30 min and 1 h. Some dipeptides are known to have physiological or cell-signaling effects, although most of them are simple short-lived intermediates on their way to specific amino acid degradation pathways. All these dipeptides contained BCAAs residue. Up-regulation of these dipeptides showed another fate of BCAAs in the blood, these results are in line with the decline of leucine and isoleucine after feeding. We also found a significant increase of plasma betaine concentration at 2 h after CD intake. Betaine functions as a methyl donor in the formation of methionine [34], which can be explained why methionine content was higher in 4 h than 1 h. The levels of four identified metabolites, linoleic acid, 8(*R*)-hydroperoxylinoleic acid, 13-*L*-hydroperoxylinoleic acid and bovinic acidwere significantly up-regulated after CD intake. These metabolites were all related to linoleic acid metabolism pathway. 13-*L*-Hydroperoxylinoleic acid [13(*S*)-HPODE] is one of the primary products of the major polyunsaturated fatty acids (linoleic acid and arachidonic acid) [35]. Among the identified compounds of current studies, sphingosine, *L*-serine and 3-dehydrosphinganine were participated in sphingolipid metabolism. Triglycerides, phospholipids and sphingolipids were all involved in lipid metabolism as well. Most of these metabolites showed significant increase after CD intake than PD, indicating the main changes of postprandial metabolites after CD intake were enhanced sphingolipid and lipid metabolism.

## Conclusion

Based on biochemical parameters and metabolomics results, a broad map of how postprandial metabolites changed over time after intake of PD and CD was shown. Plasma glucose and most amino acid showed a significant increase while plasma triglycerides, glutamate and glycine were lower after intake of both diets. The metabolomics results showed metabolites such as 5-aminopentanoic acid, pipecolic acid, ornithine and 5-hydroxy-*L*-tryptophan were significantly increased in plasma during the first hour, whereas the concentrations of plasma triglycerides, glutamate, glycine, palmitelaidic acid, 13-HODE, and oleic acid were decreased. The down-regulation of some lipid-related metabolites at 1 h postprandially were consistent with the lower concentration of plasma triglyceride, while dipeptides were up-regulated and concentration of plasma leucine and isoleucine declined in CD. In summary, these findings provide better knowledge of postprandial nutritional biochemistry and physiology and are of great importance for further nutritional intervention strategies.

## Additional file

Additional file 1: **Table S1.** The results of biochemical analysis between PD^a^ and CD^b^. (DOCX 25 kb)

Additional file 2: **Table S2.** List of identified metabolites over time of PD and CD. (DOCX 21 kb)

## Abbreviations

13-HODE: 13-Hydroxyoctadecadienoic acid
AA: Amino acids
ALB: Albumin
BCAAs: Branched chain amino acids
CD: A common corn-soybean meal diet
CREA: Creatinine
EAA: Essential free amino acids
HDL: High density lipoprotein
HPODE: Hydroperoxylinoleic acid
LDL: Low density lipoprotein
LysoPC: Lysophosphatidylcholines
LysoPE: Lysophosphatidylethanolamine
PD: A starch-casein based purified diet
TAA: Total free amino acids
UPLC-MS: Ultra performance liquid chromatograph-mass spectrometer
