# Supplementary material for: Dynamic changes of postprandial plasma metabolites after intake of corn-soybean meal or casein-starch diets in growing pigs
Source: J Anim Sci Biotechnol. 2019 May 28;10:48. doi: 10.1186/s40104-019-0351-8 (PMC6542062; doi:10.1186/s40104-019-0351-8)
Supplement: Supplementary file 1 — Table S1. The results of biochemical analysis between PDa and CDb. (DOCX 25 kb) [file 40104_2019_351_MOESM1_ESM.docx]

Table S1. The results of biochemical analysis between PD^a^ and CD^b^.

| Time spots | ALB^c^ , g/L | | | |  | PP^d^ , mmol/L | | | |  | HDL^e^ , mmol/L | | | |  | LDL^f^ , mmol/L | | | |  | CREA^g^ , mmol/L | | | |
| --- | --- | --- | --- | --- | --- | --- | --- | --- | --- | --- | --- | --- | --- | --- | --- | --- | --- | --- | --- | --- | --- | --- | --- | --- |
|  | PD | CD | SEM | *P-*value |  | PD | CD | SEM | *P-*value |  | PD | CD | SEM | *P-*value |  | PD | CD | SEM | *P-*value |  | PD | CD | SEM | *P-*value |
| 0 | 35.21 | 35.18 | 0.75 | 0.31 |  | 2.55 | 2.34 | 0.41 | 0.65 |  | 0.48 | 0.47 | 0.02 | 0.82 |  | 1.61 | 1.45 | 0.21 | 0.45 |  | 68.85 | 75.45 | 12.31 | 0.83 |
| 0.5 | 35.9 | 36.16 | 1.21 | 0.26 |  | 2.63 | 2.62 | 0.08 | 0.75 |  | 0.50 | 0.50 | 0.03 | 0.91 |  | 1.59 | 1.59 | 0.04 | 0.97 |  | 72.4 | 69.06 | 4.62 | 0.76 |
| 1 | 36.43 | 35.96 | 2.26 | 0.78 |  | 2.46 | 2.60 | 0.21 | 0.73 |  | 0.50 | 0.48 | 0.03 | 0.82 |  | 1.59 | 1.62 | 0.02 | 0.71 |  | 74.77 | 71.54 | 5.21 | 0.54 |
| 2 | 37.02 | 37.49 | 0.96 | 0.82 |  | 2.50 | 2.57 | 0.12 | 0.52 |  | 0.52 | 0.50 | 0.02 | 0.76 |  | 1.65 | 1.72 | 0.08 | 0.65 |  | 79.16 | 76.88 | 6.48 | 0.69 |
| 4 | 35.22 | 36.95 | 1.82 | 0.54 |  | 2.42 | 2.49 | 0.09 | 0.21 |  | 0.50 | 0.48 | 0.04 | 0.63 |  | 1.56 | 1.69 | 0.15 | 0.68 |  | 71.49 | 74.63 | 5.24 | 0.68 |
| 8 | 36.66 | 36.87 | 0.33 | 0.56 |  | 2.31 | 2.18 | 0.18 | 0.17 |  | 0.49 | 0.47 | 0.04 | 0.45 |  | 1.55 | 1.63 | 0.23 | 0.88 |  | 72.23 | 72.28 | 0.58 | 0.89 |

^a^ PD: a starch-casein based purified diet. ^b^ CD: a common corn-soybean meal diet. ^C^ ALB: albumin. ^d^ PP: plasma phosphorus. ^e^ HDL: High-density lipoprotein cholesterol. ^f^ LDL: Low-density lipoprotein cholesterol. ^g^ CREA: creatinine
