# Supplementary material for: Dynamic changes of postprandial plasma metabolites after intake of corn-soybean meal or casein-starch diets in growing pigs
Source: J Anim Sci Biotechnol. 2019 May 28;10:48. doi: 10.1186/s40104-019-0351-8 (PMC6542062; doi:10.1186/s40104-019-0351-8)
Supplement: Supplementary file 2 — Table S2. List of identified metabolites over time of PD and CD. (DOCX 21 kb) [file 40104_2019_351_MOESM2_ESM.docx]

Supplementary table 2 List of identified metabolites over time of PD and CD diets.

| Name | RT^a^ | MS^b^ |
| --- | --- | --- |
| (3S,5S) -3,5-Diaminohexanoate | 0.79 | 146.1876 |
| Ornithine | 0.80 | 132.1610 |
| L-Lysine | 0.80 | 146.1876 |
| Glycerophosphocholine | 0.85 | 257.2213 |
| L-2-Amino-5-hydroxypentanoic acid | 0.92 | 133.1457 |
| 4-Amino-2-methylenebutanoic acid | 0.92 | 115.1305 |
| D-Proline | 0.92 | 115.1305 |
| 4-Amino-3-hydroxybutyrate | 1.04 | 119.1192 |
| (2R,3R,4R) -2-Amino-4-hydroxy-3-methylpentanoic acid | 1.05 | 147.1723 |
| L-Valine | 1.05 | 117.1463 |
| 5-Aminopentanoic acid | 1.05 | 117.1463 |
| 2-Pentenoic acid | 1.05 | 100.1158 |
| Pipecolic acid | 1.11 | 129.1570 |
| 2-Pentenoic acid | 1.21 | 100.1158 |
| 3-Aminocaproic acid | 1.43 | 131.1729 |
| L-gamma-glutamyl-L-valine | 1.83 | 246.2630 |
| L-beta-aspartyl-L-leucine | 1.83 | 246.2603 |
| Glu-Val | 1.83 | 246.2603 |
| Aspartyl-Leucine | 1.83 | 246.2603 |
| Aspartyl-Isoleucine | 1.83 | 246.2603 |
| Cinnamic acid | 1.95 | 148.1586 |
| 4-Methylcatechol | 1.95 | 124.1372 |
| Serinyl-Gamma-glutamate | 2.55 | 233.2218 |
| L-leucyl-L-proline | 2.55 | 228.2881 |
| Indoleacrylic acid | 2.65 | 187.1947 |
| 5-Hydroxy-L-tryptophan | 2.66 | 220.2246 |
| L-gamma-glutamyl-L-leucine | 2.83 | 260.2869 |
| L-gamma-glutamyl-L-isoleucine | 2.83 | 260.2900 |
| Methylparaben | 4.24 | 152.1473 |
| Palmitelaidic acid | 8.65 | 254.4082 |
| 5-Hexyltetrahydro-2-furanoctanoic acid | 8.68 | 298.4608 |
| Phytosphingosine-1-P | 8.89 | 397.4871 |
| LysoPE (0:0/18:1 (9Z)) | 9.29 | 479.5876 |
| 3-Methyl-5-pentyl-2-furannonanoic acid | 9.34 | 308.4556 |
| LysoPE (0:0/20:4 (8Z,11Z,14Z,17Z)) | 9.42 | 501.5931 |
| (Z)-15-Oxo-11-eicosenoic acid | 9.58 | 324.4980 |
| 13-L-Hydroperoxylinoleic acid | 9.79 | 312.4443 |
| 13-HOTE | 9.79 | 294.4290 |
| 9-HOTE | 9.79 | 294.4290 |
| N, O-Didesmethyltramadol | 9.94 | 235.3220 |

Continue Supplementary table 2

| Name | RT^a^ | MS^b^ |
| --- | --- | --- |
| (9S,10E,12Z,15Z) -9-Hydroxy-10,12,15-octadecatrienoic acid | 9.98 | 294.4290 |
| LysoPC (22:4 (7Z,10Z,13Z,16Z)) | 10.08 | 571.7260 |
| 3alpha-Hydroxyoreadone | 10.08 | 252.3062 |
| 2-Hydroxyacorenone | 10.09 | 236.3499 |
| Pentadecanoylglycine | 10.24 | 299.4488 |
| Octadecanedioic acid | 10.25 | 314.4602 |
| Linoleic acid | 10.37 | 280.4455 |
| LysoPC (22:5 (4Z,7Z,10Z,13Z,16Z)) | 10.45 | 571.7260 |
| 2,4-Heptadecanedione | 10.50 | 268.4348 |
| Phytosphingosine | 10.76 | 317.5072 |
| 12-Hydroxy-8,10-octadecadienoic acid | 10.92 | 296.4449 |
| 13-HODE | 10.92 | 296.4449 |
| 9-HODE | 10.92 | 296.4449 |
| Sphingosine | 10.99 | 299.4919 |
| 3-Dehydrosphinganine | 10.99 | 299.4919 |
| 5,10-Pentadecadien-1-ol | 11.01 | 224.3822 |
| Vaccenic acid | 11.04 | 282.4680 |
| Oleic acid | 11.04 | 282.4614 |
| 18-Hydroxyarachidonic acid | 11.10 | 320.4663 |
| (ent-16alpha) -16-Kauranol | 11.59 | 290.4834 |
| (3S,6E,10E) -1,6,10,14-Phytatetraen-3-ol | 11.59 | 290.4834 |
| Bovinic acid | 11.77 | 280.4455 |
| Eicosenoic acid | 11.87 | 310.5145 |
| L-Serine | 13.27 | 105.0926 |

^a^ M-to-Z ratio. ^b^ retention time
